# Taohong Siwu Decoction Regulates Cell Necrosis and Neuroinflammation in the Rat Middle Cerebral Artery Occlusion Model

**DOI:** 10.3389/fphar.2021.732358

**Published:** 2021-08-10

**Authors:** Ni Wang, Changyi Fei, Furui Chu, Shi Huang, Lingyu Pan, Daiyin Peng, Xianchun Duan

**Affiliations:** ^1^ School of Pharmacy, Anhui University of Chinese Medicine, Hefei, China; ^2^ Department of Pharmacy, The First Affiliated Hospital of Anhui University of Traditional Chinese Medicine, Hefei, China

**Keywords:** taohong siwu decoction, ischemic stroke, cell death, nerve inflammation, rats

## Abstract

Cell necrosis and neuroinflammation play an important role in brain injury induced by ischemic stroke. Previous studies reported that Taohong Siwu decoction (THSWD)can reduce heart muscle cell necrosis and has anti-inflammatory properties. In this study, we investigated the effects of THSWD on cell necrosis and neuroinflammation in a rat model of middle cerebral artery occlusion (MCAO). Thirty-six male Sprague-Dawley (SD) rats were randomly divided into three groups with 12 rats in each group. They were the sham operation group, MCAO model group, and MCAO + THSWD group. We used ELISA to determine the levels of TNF-α, Mcp-1, and IL-1β inflammatory factors in rat serum, qRT‐PCR to detect the expression of TNF‐α, Mcp‐1 and IL‐1β mRNA in rat brain, and immunohistochemistry to detect the number of microglia and neutrophils in rat brain. qRT-PCR and Western blot were used to detect the mRNA and protein expression levels of IBA-1 and MPO inflammatory factors and the TNF-α/RIP1/RIP3/MLKL pathway in the rat brain and protein expression levels. Compared with the sham operation group, the expression of MCP-1, IL-1β, IBA-1, and MPO inflammatory factors and the TNF-α/RIP1/RIP3/MLKL pathway were significantly upregulated in the MCAO group. Compared with the MCAO group, the expressions of MCP-1, IL-1β, IBA-1, and MPO inflammatory factors and the TNF-α/RIP1/RIP3/MLKL pathway were significantly downregulated in the MCAO + THSWD group. THSWD can reduce the expression levels of MCP-1, IL-1β, IBA-1, and MPO inflammatory factors as well as the TNF-α/RIP1/RIP3/MLKL pathway. Meanwhile, it can reduce the necrosis and inflammation of brain cells after cerebral ischemia, so as to protect the brain tissue of rats.

## Introduction

Ischemic stroke is a disorder of cerebral blood circulation, which causes cerebral ischemia and hypoxia and leads to localized ischemic necrosis of brain tissue. Meanwhile, it can activate nonspecific inflammation of local tissue, which is an important factor for aggravating ischemic brain injury. Cell necrosis and neuroinflammation play an important role in transient ischemic stroke and reperfusion (Chen et al., 2016; Wang et al., 2021). For example, the neuroinflammatory response to ischemic stroke is characterized by astrocyte activation, microglial residence, infiltration of peripheral leukocytes, and release of pro-inflammatory mediators. In addition, infiltrating neutrophils and activated microglia produce free radicals and oxidants that damage central nervous system tissues, which lead to long-term disability and death in stroke patients (Roger et al., 2012). Therefore, the development of a protective strategy against cell necrosis and neuroinflammation may be an effective approach in the treatment of patients with ischemic stroke.

Taohong Siwu Decoction (THSWD) is from Wu Qian’s “The Golden Guide to Medicine” of the Qing Dynasty. The formula consists of safflower and peach seeds, which promote blood circulation and remove blood stasis. Meanwhile, it also uses angelica and rehmanniae to replenish qi and produce blood (Zhang, 2014). Studies have shown that THSWD have many advantages for dong *s*th. For example, inhibiting the expression of pro-apoptotic protein Bax, promoting the expression of anti-apoptotic protein Bcl2, reducing the exudation of lactate dehydrogenase (LDH), increasing the activity of antioxidant enzyme superoxide dismutase (SOD), and reducing the content of lipid oxide malondialdehyde (MDA) in a simulated ischemic animal model, etc. The mechanism may be related to the inhibition of cell oxidation and apoptosis (Mao et al., 2016). In the rat model of postherpetic neuralgia, THSWD has an analgesic effect on postherpetic neuralgia in rats by inhibiting the release of inflammatory factors such as tumor necrosis factor-α (TNF-α) and interleukin-1β (IL-1β) and reducing the apoptosis of spinal nerve cells (Xu et al., 2021). Clinically, studies have shown that THSWD can effectively dredge the blood and qi in patients with ischemic stroke, and improve the microcirculation of the brain tissue. Especially, it can reduce the patient’s body inflammation and nerve damage, which more effectively promotes restoration of nerve function in the patient (Zhang, 2019). The research by Wang et al. (2020) shows that THSWD can decrease the activation of NLRP3 inflammasome, downregulate GSDMD, and inhibit cell pyrotosis in MCAO/R rats. Chen et al., 2020 research shows that THSWD can promote angiogenesis after cerebral ischemia in rats by regulating platelet particles, and then treating cerebral ischemia. Other studies have shown that THSWD may regulate the survival of neurons by upregulating the expression of brain-derived neurotrophic factor (BDNF), activating the BDNF-TrkB-ERK1/2 signaling pathway, there by promoting the recovery of cerebral ischemic injury (Wu, 2018).

From the perspective of apoptosis, programmed cell necrosis initiated by the tumor necrosis factor receptor (TNFR) family and toll-like receptor (TLRS) family is the most studied necrosis pathway at present. In the cell, it is mainly mediated by receptor-interacting protein kinases RIPK1 and RIPK3, recruited, and phosphorylated into a mixed lineage kinase-domain-like protein (MLKL), which eventually forms necrotic bodies and causes cell death (Shan et al., 2018).

By replicating the rat model of cerebral ischemia, this study explored whether THSWD can reduce cell necrosis and neuroinflammation in the rat model of MCAO by inhibiting the expression levels of MCP-1, IL-1β, IBA-1, and MPO inflammatory factors as well as the TNF-α/RIP1/RIP3/MLKL pathway. It provides an experimental basis and theoretical basis for the further development of THSWD.

## Materials and Methods

### Animals

A total of 36 adult male Sprague-Dawley rats (230–270 g) were obtained from the Experimental Animal Center of Anhui Medical University. Rats were kept in a humidity and temperature-controlled chamber with a 12-h light/dark cycle and free access to water and food.

### Medicinal Materials

THSWD composition: angelica (Angelicae Sinensis Radix, batch number: 16070501), rehmanniae (Rehmanniae Radix Praeparata, batch number: 17042501) and chuanxiong (Chuanxiong Rhizoma, batch number: 17061601), radix paeoniae alba (Paeoniae Radix Alba, batch number: 17050301), peach kernel (Persicae Semen, batch number: 17033101), safflower (Carthami Flos, batch number: 17041401) each 100 g, All purchased from the pharmacy of the First Affiliated Hospital of Anhui University of Traditional Chinese Medicine. According to the ratio of peach kernel: safflower: rehmanniae: angelica: radix paeoniae alba: chuanxiong = 3:2:4:3:3:2, put the herbal medicine in a beaker, first extract with 10 times the amount of water for 2 h, filter, save the filtrate, the filtrate is extracted with 8 times the amount of water for 1.5 h, filter, combine the two filtrates, concentrate the filtrate to make a concentrate containing 0.9 g/ml of herbal medicine, and store it in a low temperature place away from light (Duan et al., 2020).

### Animal Groups

After adaptive feeding for 1 week, 36 rats were randomly divided into three groups with 12 mice in each group: sham operation group, MCAO group and MCAO + THSWD group. In the MCAO + THSWD group, 9 g/(kg-d) of THSWD was administered by gavage on the second day of modeling, and the same amount of saline was administered by gavage on the second day of modeling in the MCAO and sham operation groups once a day for 7 days. All rats were anesthetized by intraperitoneal injection of chloral hydrate (350 mg-kg) 2 h after the last dose, and the brain tissues were removed by severing the head, and the brain tissues of each group were fixed with 4% paraformaldehyde and frozen at −80°C in the refrigerator.

### Preparation of MCAO

The model of middle cerebral artery occlusion (MCAO) in rats was established according to the method of Longa et al. (Longa et al., 1989). After fasting for 12 h before surgery, the rats were anesthetized by intraperitoneal injection of 10% chloral hydrate (350 mg/kg^−1^). The right common carotid artery, external carotid artery, and internal carotid artery were successively separated. The main external carotid artery was ligated and dissociated. When the nylon thread was inserted 18–20 mm from the bifurcation of the common carotid artery and there was slight resistance, the thread was stopped. After 2 h of ischemia, the thread was gently pulled out to restore blood perfusion. The external carotid artery was ligated and the skin was sutured. In the sham operated group, only a surgical incision was made and no wire plugs were inserted. It was assessed using the Zea Longa 5-point scale (Longa et al., 1989): 0 points–no neurological deficit; 1 points–when lifting the tail, it was seen that the left front paw could not be fully extended; 2 points–when lifting the tail, it was seen that the left front paw could not be fully extended and turned in a circle to the left; 3 points–it was seen that the rat rotated or tilted to the left when walking; 4 points–it was seen that the rat could not walk spontaneously and lost consciousness. Those with scores of 1–3 were included in the experiment, and those with scores of 0 or 4 were excluded.

### ELISA

Blood was collected from the rat abdominal aorta and centrifuged at 2000 rpm/min for 15 min after 30 min at room temperature, and the serum was extracted. The relevant indexes were tested according to the Elisa kit procedure.

### Immunohistochemistry

The brain tissue fixed in 4% paraformaldehyde solution was removed for paraffin-embedding and made into paraffin sections. The procedure was as follows: Dewaxing → hydration → antigen repair → normal serum sealing → drop addition of MPO and IBA-1 antibodies anti-Iba1 (abclonal, A1527, 1:100) and anti-Mpo (abclonal, A1374, 1:100) → DAB color rendering → redyeing → blue return → gradient alcohol dehydration → transparent sealing → the number and morphology of microglia and neutrophils were analyzed by microscopic observation.

### Quantitative Real-Time PCR

Total RNA was extracted from each group of rat brain tissue according to the instructions of EZ-10 Total RNA Mini-Preps Kit (B618583, Sangon Biotech). Quantitative PCR primers were designed according to Primer Premier version 6.0 software, and reverse transcription was performed according to ABScript II RT Mix for qPCR with gDNA Remover (RK20403, Abclonal) reverse transcription kit. qPCR reactions were performed using a Biorad IQ5 real-time PCR instrument with 2× Universal SYBR Green Fast qPCR Mix (RK21203, Abclonal) in sybr green (Supplementary Table S1).

**TABLE 1:** Primer sequence.

Primer name		Sequence (5′-3′)	Product length
Actb	F	CCT​CAC​TGT​CCA​CCT​TCC​A	120
	R	GGG​TGT​AAA​ACG​CAG​CTC​A	
Iba-1	F	GCAGCCTCATCGTCATCT	118
	R	CTC​TCT​TCC​TGT​TGG​GCT​T	
Mpo	F	CTGGCACGGAAGCTGAT	120
	R	AATGAGGCAGGCAAGGAG	
MCP-1	F	CAGGTCTCTGTCACGCTTC	148
	R	AGTTCTCCAGCCGACTCA	
IL-1β	F	CCCTTGACTTGGGCTGT	60
	R	CGAGATGCTGCTGTGAGA	
Tnf-α	F	CAGCCAGGAGGGAGAAC	93
	R	GTA​TGA​GAG​GGA​CGG​AAC​C	
RIP1	F	CATCCCACCAGACAAGGT	109
	R	CCCAGAACTCAAGAGGCA	
RIP3	F	GCATCCTTCCAAACCCA	140
	R	CGCACCATTGAGCCATA	
MLKL	F	CCCAGTCAAACTCCTCCTC	131
	R	ACAATACCCCACCACACC	

### Western Blot

The rats were treated with the above method by Western blot. The left brain tissue was taken and put into a 5 ml enzyme-free centrifuge tube. Magnetic beads and RIPA solution containing PMSF were added, and the tissue was smashed with a tissue grinding instrument. The nuclear proteins were extracted according to the instructions of the nuclear protein and cytoplasmic protein extraction kit. According to the instructions of BCA Protein Assay Kit, determine the protein concentration of the sample, make a standard curve, calculate the protein concentration, add the corresponding SDS Protein Loading Buffer, mix well and heat in a metal bath thermostat at 100°C for 10 min, and place on ice. The proteins were separated by SDS-PAGE gel electrophoresis, then transferred to PVDF membrane and closed at room temperature for 2 h using 5% skimmed milk powder. The PVDF membrane was added to TBST and washed 3 times for 8 min each. Add the corresponding primary antibody, the membrane was incubated overnight at 4°C with primary antibodies including anti-Actb (abclonal, AC038, 1:10000), anti-Iba-1 (abclonal, A1527, 1:500), anti-Mpo (abclonal, A1374, 1:1000), anti-Tnfa (abclonal, A0277, 1:1000), anti-Rip1 (abclonal, A7414, 1:1000), anti-Rip3 (abclonal, A12996, 1:1000), anti-Mlkl (A5579, 1:1000) and wash the membrane 3 times with TBST for 8 min each time the next day. The PVDF membranes were then incubated with the secondary antibody (HRP Goat Anti-Rabbit IgG (H+L) (abclonal, AS014, 1:8000), and developed with enhanced chemiluminescence (ECL, beyotime, P0018AM). The PVDF membrane was put into the ultra-sensitive multifunctional imager, the developing solution was added, and the bands with different objectives were obtained by exposure after the reaction, and the protein grayscale values were analyzed by Image J software.

### Statistical Methods

SPSS 20.0 software was used to statistically analyze the data. Normally distributed measures were expressed as mean ± standard deviation (x ¯± s), and independent samples t-test was used for comparison between groups, paired t-test for comparison within groups, and one-way ANOVA was used for comparison of data between multiple groups. *p* < 0.05 was considered a statistically significant difference.

## Results

### Neurological Deficit Score

**FIGURE 1:**
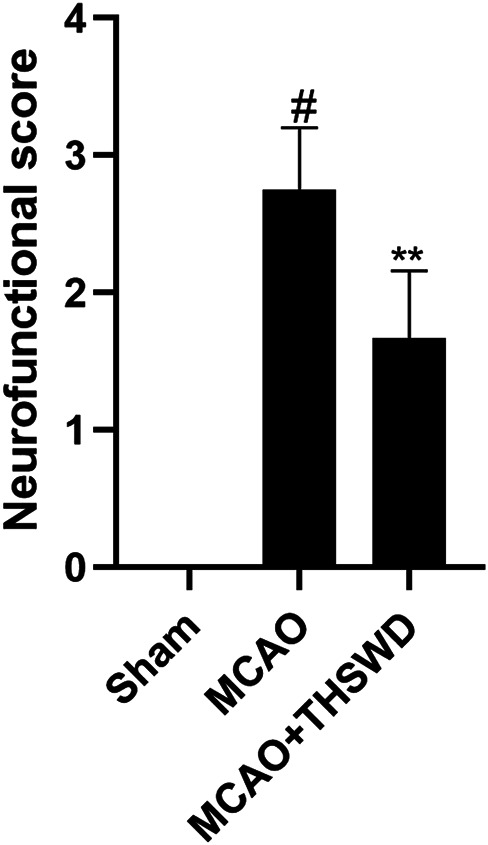
Neurological score. Notes: #*P*<0.01 vs Sham operation group; **P<0.05 vs MCAO group.

Compared with the sham operation group, the neurological deficit score was significantly higher in the MCAO group (*p* < 0.01), indicating successful modeling, and the neurological deficit score was significantly lower in the MCAO+THSWD group compared with the MCAO group (*p* < 0.05), as shown in Figure 1.

### THSWD Inhibited TNF-α, MCP-1, and IL-1β

**FIGURE 2:**
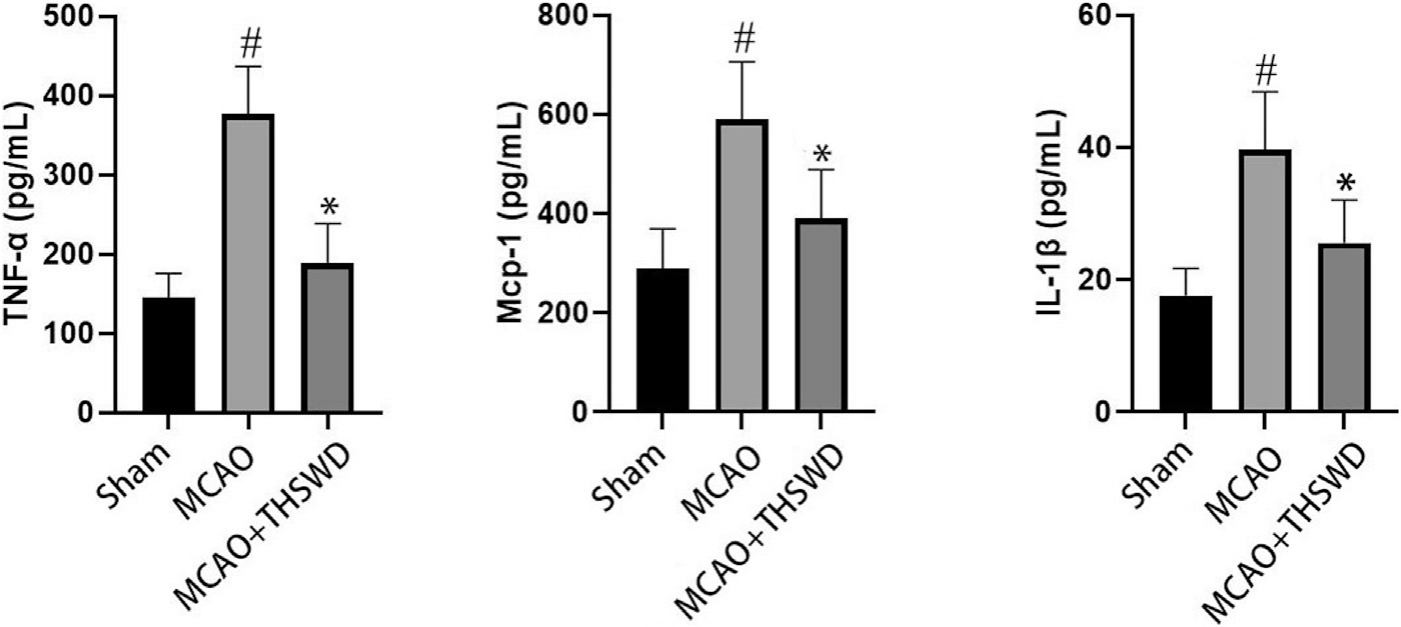
TNF-α, MCP-1, and IL-1β were detected by ELISA. Notes: #*P*<0.01 vs Sham operation group; *P<0.01 vs MCAO group.

**FIGURE 3:**
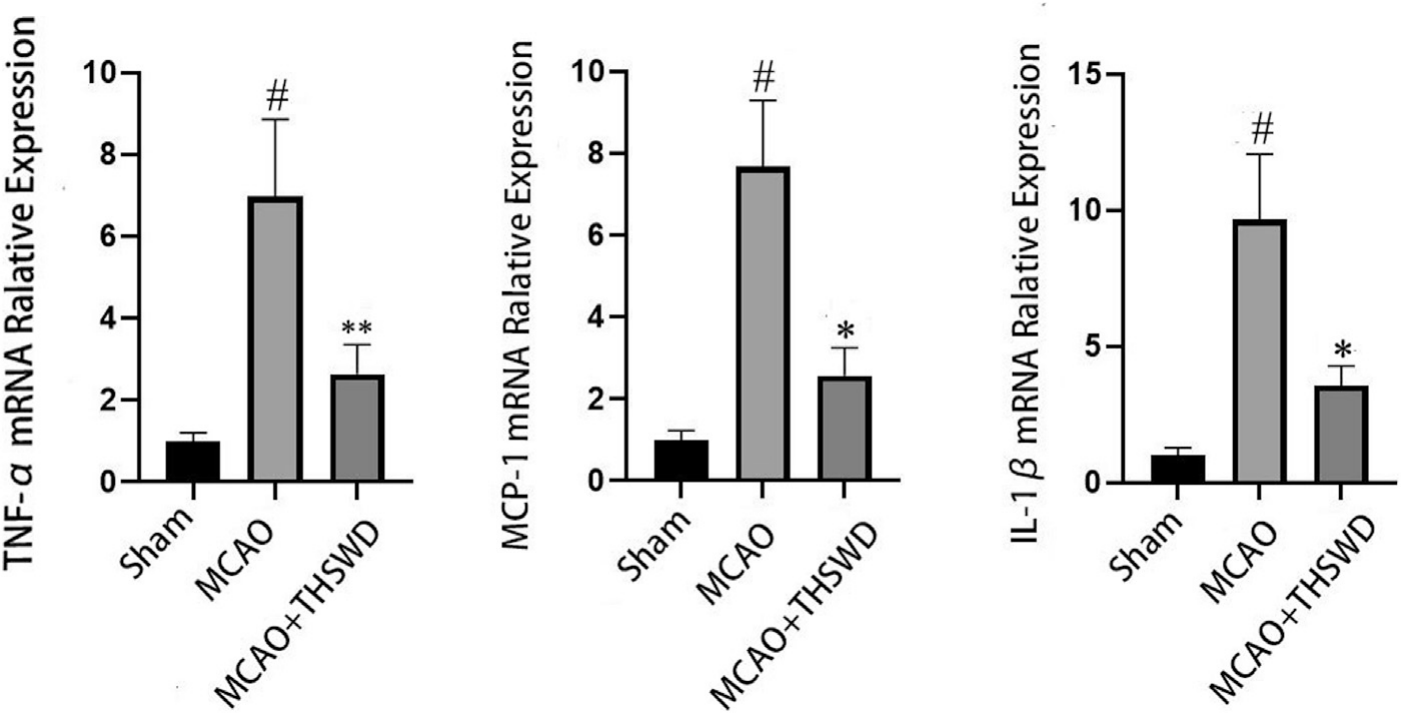
mRNA expression of TNF-α, MCP-1, and IL-1β. Notes: #*P*<0.01 vs Sham operation group; *P<0.01, **P<0.05 vs MCAO group.

Elisa results showed that the levels of TNF-α, Mcp-1 and IL-1β were significantly increased in the MCAO group compared with the sham operation group (*p* < 0.01), and the levels of TNF-α, Mcp-1 and IL-1β were significantly decreased in the MCAO+THSWD group compared with the MCAO group (*p* < 0.01), as shown in Figure 2. The qPCR results showed that TNF-α, Mcp-1, and IL-1β mRNA expressions were significantly increased in the MCAO group compared with the sham operation group (*p* < 0.01), and TNF-α, Mcp-1, and IL-1β mRNA expressions were significantly decreased in the MCAO+THSWD group compared with the MCAO group (*p* < 0.05, *p* < 0.01), as shown in Figure 3. It indicates that THSWD can inhibit the release of TNF-α, Mcp-1, and IL-1β inflammatory factors.

### THSWD Inhibited IBA-1 and MPO

**FIGURE 4:**
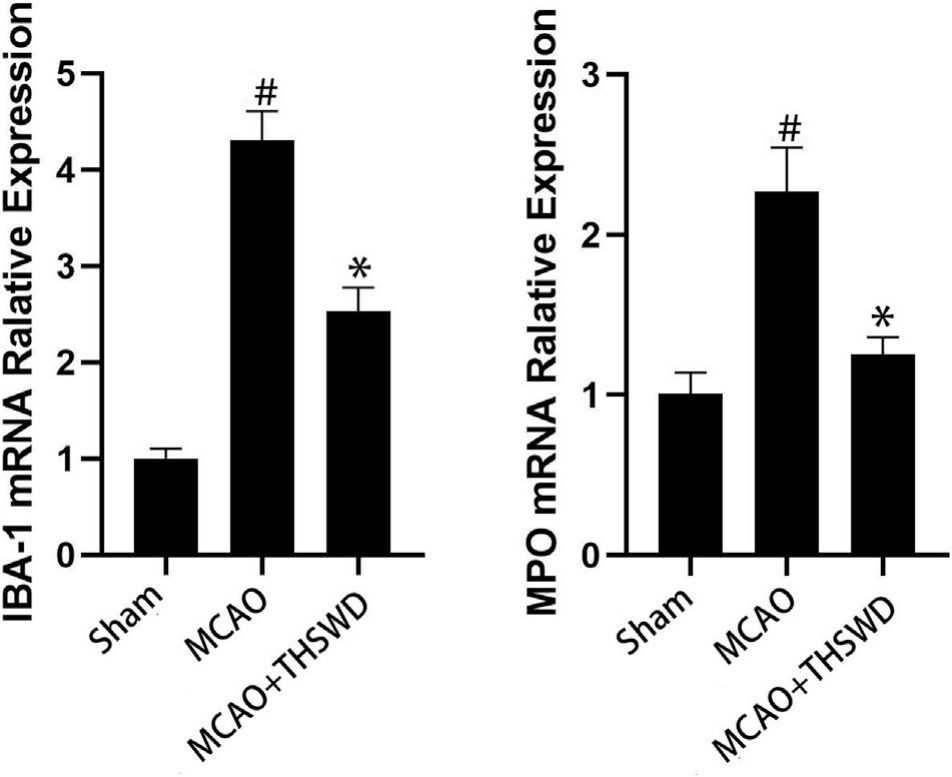
mRNA expression of IBA-1 and MPO. Notes: #*P*<0.01 vs Sham operation group; *P<0.01 vs MCAO group.

**FIGURE 5:**
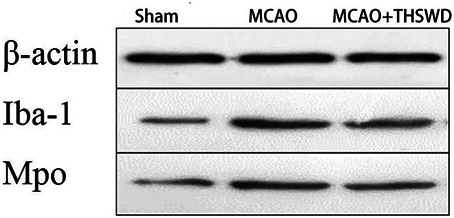
β-actin is an internal reference. The blacker the band, the higher the protein expression level.

**FIGURE 6:**
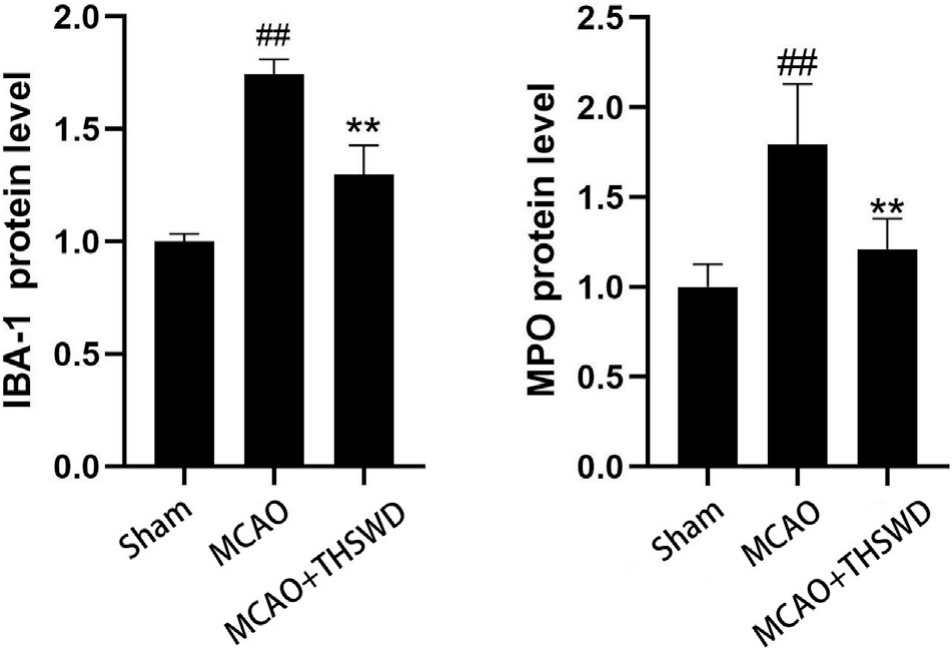
Protein expression of IBA-1 and MPO Notes: ##*P*<0.05 vs Sham operation group; **P<0.05 vs MCAO group.

IBA-1 levels are used to assess microglial activation in brain tissue (Chen et al., 2018). MPO levels are used to assess neutrophil infiltration (Yu et al., 2018.). The qPCR results showed that the mRNA expressions of IBA-1 and MPO significantly increased in the MCAO group compared with the sham operation group (*p* < 0.01); compared with MCAO group, the mRNA expression of IBA-1 and MPO in the MCAO + THSWD group decreased significantly (*p* < 0.01), as shown in Figure 4. The results of Western blot showed that the protein expression of IBA-1 and MPO in the MCAO group significantly increased compared with the sham operation group (*p* < 0.05). Compared with the MCAO group, the expression of IBA-1 and MPO protein in the MCAO + THSWD group decreased significantly (*p* < 0.05), as shown in Figures 5, 6.

**FIGURE 7:**
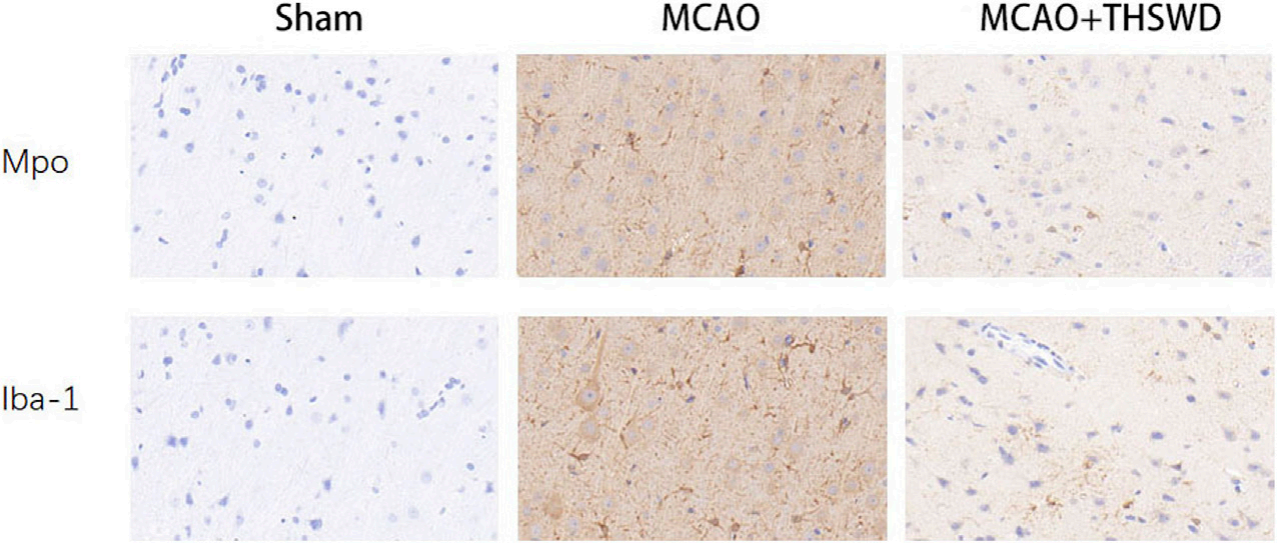
Expression of microglia and neutrophils in each group.

We next evaluated the activation status of microglia and the degree of neutrophil infiltration in brain tissue using IBA-1 and MPO. Immunohistochemical results (Figure 7) showed that microglia and neutrophils were mostly in a resting state in normal conditions; microscopic observation revealed that microglia and neutrophils were heavily activated and infiltrated in the ischemic parts of the brain of rats in the MCAO group, with increased numbers and larger cytosomes. There was an increased number of microglia and neutrophils in the same part of the rat brain compared to the sham operated group. After administration of THSWD treatment to MCAO model rats, microglia and neutrophil activation and infiltration at the ischemic site of the rat brain were significantly inhibited, and the number of IBA-1 and MPO positive cells was significantly reduced compared with the MCAO group. The results indicate that THSWD can inhibit the hyperactivation and infiltration of microglia and neutrophils at the ischemic site in the brain of MCAO model rats.

### THSWD Inhibited the TNF-α/RIP1/RIP3/MLKL Pathway

**FIGURE 8:**
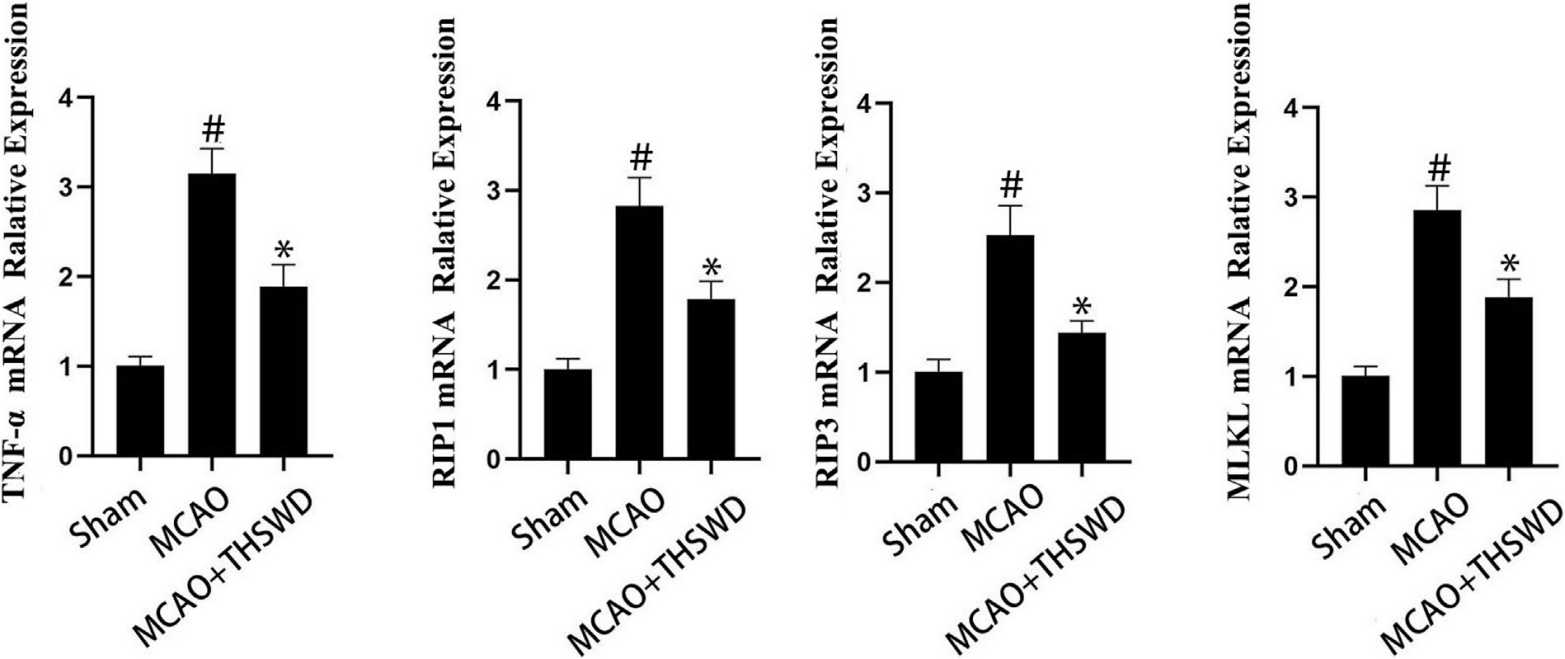
mRNA expression of TNF-α/RIP1/RIP3/MLKL. Notes: #*P*<0.01 vs Sham operation group; *P<0.01 vs MCAO group.

**FIGURE 9:**
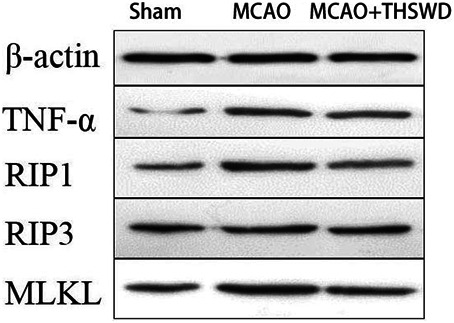
β-actin is an internal reference. The blacker the band, the higher the protein expression level.

**FIGURE 10:**
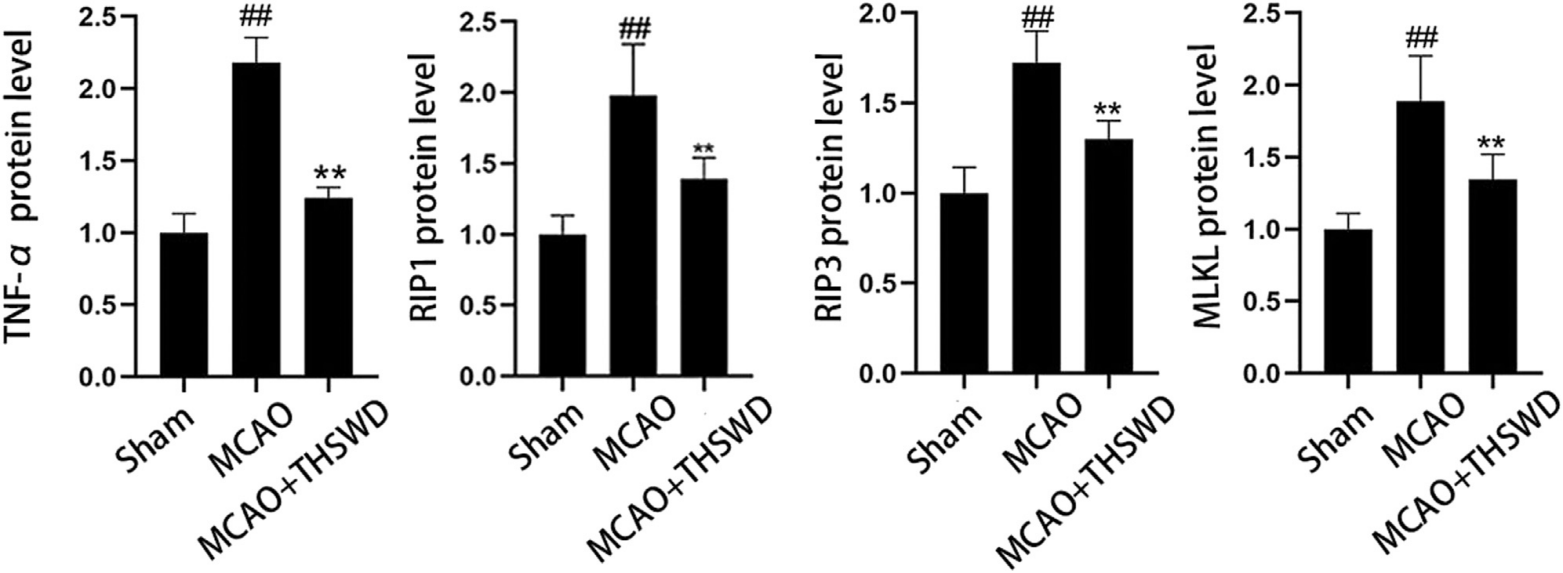
Protein expression of TNF-α/RIP1/RIP3/MLKL Notes: ##*P*<0.05 vs Sham operation group; **P<0.05 vs MCAO group.

TNF level is used to evaluate the expression of cellular inflammatory factors (Zhao and Zhang, 2021). The RIP1-RIP3-MLKL pathway is used to evaluate programmed apoptosis (Zhang et al., 2020). The qPCR results showed that the mRNA expression of TNF-α/RIP1/RIP3/MLKL significantly increased in the MCAO group compared with the sham operation group (*p* < 0.01); compared with the MCAO group, the mRNA expression of TNF-α/RIP1/RIP3/MLKL in the MCAO + THSWD group significantly decreased (*p* < 0.01), as seen in Figure 8. Western blot results showed that compared with the sham operation group, the expression of TNF-α/RIP1/RIP3/MLKL proteins significantly increased in the MCAO group (*p* < 0.05); compared with the MCAO group, the protein expression of TNF-α/RIP1/RIP3/MLKL in the MCAO + THSWD group significantly decreased (*p* < 0.05), seen in Figures 9, 10.

## Discussion

In this study, we demonstrated that THSWD alleviated cell necrosis and neuroinflammation after MCAO in rats. The following observations were made: 1) THSWD inhibited neutrophil infiltration, microglia activation, and cell necrosis in the ischemic area after MCAO; 2) by inhibiting the expression levels of MCP-1, IL-1β, IBA-1, and MPO inflammatory factors and the TNF-α/RIP1/RIP3/MLKL pathway, the anti-inflammatory effect of THSWD was promoted and the release of TNF-α, IL-6, and other inflammatory factors was reduced. In summary, our results suggest that THSWD alleviates cell necrosis and neuroinflammation in MCAO by reducing the expression levels of MCP-1, IL-1β, IBA-1, and MPO inflammatory factors and inhibiting the TNF-α/RIP1/RIP3/MLKL pathway.

More and more studies have shown that a variety of inflammatory cytokines, such as TNF-α, IL-1β, and IL-6, are released after cerebral ischemia, which can transform ischemic injury into inflammatory injury and promote the apoptosis and necrosis of nerve cells and the formation of brain edema (Fann et al., 2013). The inhibition of early inflammatory response can prevent further brain necrosis and improve cerebral nerve function (Duan et al., 2020). MPO is a marker of neutrophils activation, and the detection of MPO in brain tissue can reflect the degree of neutrophils infiltration and local inflammatory reaction (Zhang et al., 2017). It can effectively reduce the contents of TNF-α and MPO in brain tissue of rats with cerebral ischemia-reperfusion injury, which has a significant effect on brain protection (Lei et al., 2016). IBA-1 can specifically bind to microglia cells and is often used as a marker to identify microglia cells. When microglia cells are activated by chronic stress, their morphology changes and the expression of IBA-1 is upregulated (Waller et al., 2019). Consistent with these findings, THSWD reduced neutrophilic infiltration, microglia activation, and the amount of cell necrosis according to our results. Specifically, THSWD decreased and inhibited the protein expression of TNF-α (a marker of inflammation) and IBA-1 (a marker of microglia).

According to the literature, MCP-1 is a glycoprotein regulated by nuclear transcription factor-κB (NF-κB), which can be produced by mesangial cells stimulated by TNF-α (Sheryanna et al., 2007). Glycoprotein MCP-1 regulated by NF-κB not only promotes the migration, adhesion, and aggregation of inflammatory cells, but also has dual functions of inducing chemotaxis and activating monocytes to inflammatory sites (Chen et al., 2012). Chemokine ligand 1 (CXCL1), also known as GRO-α oncogene, is a member of the chemokine family (Cecchinato and Uguccioni, 2018). Originally found in melanoma, it is also expressed in macrophages, neutrophils, and epithelial cells. CXCL1 is mainly expressed and secreted by vascular endothelial cells, activated macrophages, and fibroblasts in peripheral tissues, while it is mainly expressed and secreted by neurons, microglia, oligodendrocytes, and activated astrocytes in brain tissues (Zhang et al., 2017). By recruiting neutrophils, CXCL1 activates neutrophils FGR and HCK (a member of the protein tyrosine kinase PTK family) and promotes the release of VEGF-A, thereby promoting angiogenesis *in vivo* (Scapini et al., 2004). The results of ELISA and qPCR showed that THSWD could inhibit the release of TNF-α, MCP-1, and IL-1β inflammatory factors.

Currently, there are many ways to induce programmed necrosis. Among these ways, the RIP1/RIP3/MLKL signaling pathway is the most typical one (Wang et al., 2019). RIP1/RIP3 is a key regulatory node of programmed necrosis, which is widely present in neurons (Zhou et al., 2017). Under the stimulation of death signal TNF and other stimulation, programmed cell death can be promoted through autophosphorylation (Xin et al., 2017; Huang et al., 2018). The expression level of RIP1/RIP3 was positively correlated with programmed cell necrosis. Inflammatory factors such as TNF-α and IL-6 are highly expressed in acute cerebral infarction, presenting as non-infectious inflammation (Fu et al., 2015). RIP1/RIP3 plays a key regulatory role in the radiation damage of central nervous tissue and inflammatory response of nervous tissue after vasospasm and ischemia (Das et al., 2016). MLKL is a key protein signaling molecule downstream of RIP3 in the TNF-α receptor-mediated programmed cell necrosis pathway. MLKL is a substrate for RIPK3 kinase in an inactive form. The C-terminal region of MLKL contains the kinase domain, and the N-terminal region contains the 4-helix domain. Activation of MLKL kinase depends on RIPK3-induced residual phosphorylation of the kinase domain (Qian and Sun, 2021). When phosphorylated, RIPK3 binds to MLKL’s 4HBD, thereby transforming MLKL from an inactive monomer structure to an active oligomer structure. Activated RIPK1 and RIPK3 recruit and phosphorylate MLKL and activate downstream signal transduction pathways to perform programmed cell necrosis (Zhao et al., 2012). Activation of MLKL kinase is considered to be an important link in the execution of programmed cell necrosis (Fang et al., 2019). According to the results of this paper, it can be obtained that THSWD can reduce brain injury after MCAO in rats by inhibiting the TNF-α/RIP1/RIP3/MLKL pathway and inhibiting programmed cell necrosis.

Our study showed that THSWD could reduce cell necrosis and neuroinflammation by inhibiting the expression levels of MCP-1, IL-1β, IBA-1, and MPO inflammatory factors as well as inhibiting the TNF-α/RIP1/RIP3/MLKL pathway. However, our results do not completely rule out the alternative pathways that regulate the inflammasome pathway. Therefore, further studies need to investigate the relationship of other inflammatory activators to rule out or include alternative pathways.

## Conclusion

In conclusion, our results suggest that THSWD can inhibit neutrophil infiltration, microglia activation, and cell necrosis in the ischemic area after MCAO. Meanwhile, it can inhibit the expression levels of MCP-1, IL-1β, IBA-1, and MPO inflammatory factors, and inhibit the TNF-α/RIP1/RIP3/MLKL pathway. Finally, it can reduce cell necrosis and neuroinflammation. This study supports continued research where THSWD is used as a potential treatment for patients with ischemic stroke.

## Data Availability

The raw data supporting the conclusions of this article will be made available by the authors, without undue reservation.
